# Supporting Clinicians to Use Technology to Deliver Highly Personalized and Measurement-Based Mental Health Care to Young People: Protocol for an Evaluation Study

**DOI:** 10.2196/24697

**Published:** 2021-06-14

**Authors:** Henriette C Dohnt, Mitchell J Dowling, Tracey A Davenport, Grace Lee, Shane P Cross, Elizabeth M Scott, Yun Ju C Song, Blake Hamilton, Samuel J Hockey, Cathrin Rohleder, Haley M LaMonica, Ian B Hickie

**Affiliations:** 1 Brain and Mind Centre University of Sydney Camperdown Australia

**Keywords:** mental health service delivery, youth mental health, model of care coordination, transdiagnostic, health information technology, education, training, adoption into clinical practice, Kirkpatrick evaluation

## Abstract

**Background:**

Australia’s mental health care system has long been fragmented and under-resourced, with services falling well short of demand. In response, the World Economic Forum has recently called for the rapid deployment of smarter, digitally enhanced health services to facilitate effective care coordination and address issues of demand. The University of Sydney’s Brain and Mind Centre (BMC) has developed an innovative digital health solution that incorporates 2 components: a highly personalized and measurement-based (data-driven) model of youth mental health care and a health information technology (HIT) registered on the Australian Register of Therapeutic Goods. Importantly, research into implementation of such solutions considers education and training of clinicians to be essential to adoption and optimization of use in standard clinical practice. The BMC’s Youth Mental Health and Technology Program has subsequently developed a comprehensive education and training program to accompany implementation of the digital health solution.

**Objective:**

This paper describes the protocol for an evaluation study to assess the effectiveness of the education and training program on the adoption and optimization of use of the digital health solution in service delivery. It also describes the proposed tools to assess the impact of training on knowledge and skills of mental health clinicians.

**Methods:**

The evaluation study will use the Kirkpatrick Evaluation Model as a framework with 4 levels of analysis: Reaction (to education and training), Learning (knowledge acquired), Behavior (practice change), and Results (client outcomes). Quantitative and qualitative data will be collected using a variety of tools, including evaluation forms, pre- and postknowledge questionnaires, skill development and behavior change scales, as well as a real-time clinical practice audit.

**Results:**

This project is funded by philanthropic funding from Future Generation Global. Ethics approval has been granted via Sydney Local Health District’s Human Research Ethics Committee. At the time of this publication, clinicians and their services were being recruited to this study. The first results are expected to be submitted for publication in 2021.

**Conclusions:**

The education and training program teaches clinicians the necessary knowledge and skills to assess, monitor, and manage complex needs; mood and psychotic syndromes; and trajectories of youth mental ill-health using a HIT that facilitates a highly personalized and measurement-based model of care. The digital health solution may therefore guide clinicians to help young people recover low functioning associated with subthreshold diagnostic presentations and prevent progression to more serious mental ill-health.

**International Registered Report Identifier (IRRID):**

PRR1-10.2196/24697

## Introduction

Australia’s mental health care system has long been fragmented and under-resourced, with services falling well short of demand [1]. Mental health consumers often receive inconsistent and sporadic treatment delivered by independent services, with little collaborative decision making or multidisciplinary continuous care [2]. At particular risk are young people who do not respond to brief interventions and are unable to access continuous care [3], and those who deteriorate under care [4-6]. Coordination between crisis and ongoing care providers is also poor, with medical staff in emergency departments expressing clear learning needs in developing care plans for patients presenting with mental health problems [7]. The advent of a novel coronavirus and its resulting global COVID-19 pandemic has added additional strain to Australia’s mental health services with increasing demands for crisis support and intervention [8-11], highlighting the need for a revolution of current mental health service delivery [12].

The World Economic Forum has recently called for the “…rapid deployment of smarter, digitally-enhanced health services*…*” as a means of potentially addressing demand issues and facilitating effective care coordination [13]. The recent Australian Productivity Commission’s inquiry into mental health also recommends that technology should play a larger role by improving access to the right services at the right time [14]. Moreover, it has been proposed that digital solutions may be a viable method of delivering personalized and measurement-based care to young people, transcending the often-narrow focus on symptom or risk reduction [15]. While there are ethical, data security, and privacy challenges that must be addressed in relation to the implementation of technology-based solutions [13], it has been noted that staff resistance to change and perceived technological complexity frequently present as barriers to the uptake of health information technologies (HITs) [16]. Consequently, education and training that foster engagement and address the use of HITs in practice are essential for successful adoption and optimization of technology-enabled solutions in clinical practice [17]. This increasing need for training and upskilling of clinicians working in digitally enhanced mental health services has not been addressed in the existing literature, which has previously been limited to training specific to clinicians’ disciplines, treating specific diagnoses, or intervention modalities [18-20]. As such, there is an urgent need for education and training in the effective use of digital health solutions to meet the increase in demand for mental health services and facilitate effective care coordination across a multidisciplinary workforce.

Researchers at The University of Sydney’s Brain and Mind Centre (BMC) have developed the Youth Mental Health (YMH) and Technology Program, which adopts an innovative digital health solution to prevent further progression of mental ill-health for young people in care [21]. This digital health solution includes two key components. The first is the *BMC Youth Model*, a highly personalized (ie, treatment targeted to address individual needs) and measurement-based (ie, data-driven) model of care generated from over 10 years of longitudinal research led by the BMC to assess multiple clinical and functional domains in young people presenting for mental health care and treatment [22,23]. This cohort includes 6743 young people aged 12 to 30 years, which was analyzed to uncover initial underlying neurobiology of mental ill-health in young people [24]. Findings were reported through a supplement of research articles that explore the associations between the multiple clinical and functional domains, neurobiological measures, as well as clinical, social, and functional outcomes [22]. The BMC Youth Model explicitly aims to prevent the progression of emerging and mixed syndromes into more complex and severe forms of illness and to facilitate symptomatic and functional recovery. The second is an HIT — such as the InnoWell Platform, which is listed on the Australian Register of Therapeutic Goods — that is a customizable digital toolkit to assist assessment, monitoring, and management of mental ill-health and maintenance of well-being by collecting, storing, scoring, and reporting personal and health information back to consumers and their health professionals to promote collaborative care partnerships [25]. Though we reference the InnoWell Platform as an exemplar HIT, it is important to note that the BMC Youth Model can be facilitated via any HIT so long as its design has been guided by similar clinical and scientific concepts to provide highly personalized and measurement-based care.

The education and training program has been designed to encourage the adoption of highly personalized and measurement-based care in clinical practice, to ensure young people get the right treatment at their first point of entry into care. Specifically, the program focuses on teaching youth mental health professionals how to assess, monitor, and manage complex needs and illness pathways. The education and training program now delivers information via multimodal tools, including online seminars, case study webinars, and in-service workshops. The HIT is referred to throughout the training, as it aids in facilitating highly personalized and measurement-based care (including assessment of clinical stage, pathophysiological mechanisms, and multidimensional needs) and supports the choice of treatment options while tracking a young person’s progress.

This paper briefly describes the education and training program and outlines a protocol for an evaluation study to assess the effectiveness of education and training on the adoption and optimization of use of the digital health solution in service delivery.

## Methods

### Education and Training Program

The education and training program has been designed to be accessed in-person or online. This has been done to provide access to standardized, high-quality training delivered by experienced clinician-researchers from the BMC. This is particularly important for reaching out to those services that would not ordinarily have access to such opportunities, such as health services in regional Australia. The program is currently comprised of online seminars, case study webinars, and in-service training workshops.

The online seminars introduce the theoretical, clinical, and scientific underpinnings of the YMH and Technology Program that provide the background knowledge necessary for clinicians to understand the digital health solution and begin to apply it to their own clinical practice. To date, 6 seminars delivered by experienced clinician researchers (authors ES and IH) have been recorded covering approximately 60 minutes each (see Textbox 1) and are freely accessible through the BMC Youth Mental Health Research YouTube Channel.

**Modules of the education and training program.**

1. A highly personalized and measurement-based model of care to manage youth mental health
2. Combining clinical stage and pathophysiological mechanisms to understand illness trajectories in young people
3. A comprehensive assessment framework for youth mental health care
4. Using the Brain and Mind Centre (BMC) Youth Model to personalize care options – right care, first time!
5. A youth mental health service delivery model to support highly personalized and measurement-based care
6. Maximizing the use of digital health solutions in youth mental health care

These online seminars are further enhanced by case study webinars*,* which detail practical application of the digital health solution and help to translate newly acquired knowledge into clinical skills [26]. Two such case study webinars spanning an hour each have been recorded to date and cover 3 cases, their initial presentation, and the tracking of their syndromes over time demonstrating the value of the digital health solution.

In-service training workshops will be delivered by BMC clinician-researchers and focus on the practical application of the BMC Youth Model into the specific youth mental health service. The workshops typically include case studies common to the participating service. These workshops can be conducted either face-to-face or online. To date, 2 regional *headspace* services in Northern New South Wales have attended in-service training workshops.

In order to encourage and reward participation in all components of the education and training program, continuing professional development points are available for psychiatrists, psychologists, mental health nurse practitioners, social workers, and occupational therapists. To further encourage engagement, additional specialized supplemental webinars (eg, applying the BMC Youth Model to understand the relationship between circadian rhythms and mental health and applying the BMC Youth Model to enhance psychological therapy for individuals with anxiety and other mood disorders) and case studies will be released, demonstrating how the BMC Youth Model can be applied to specific disorders and complex cases.

### An Evaluation Framework

The importance of a standardized and consistent framework for evaluation of education and training programs has previously been highlighted [27,28]. Evaluations often only focus on participants’ reactions and acquired knowledge and tend to neglect the assessment of behavioral change or impact on client outcomes, which are both critical factors in determining effectiveness [27].

This study will utilize the Kirkpatrick Evaluation Model, which includes 4 levels of training evaluation including: Reaction, Learning, Behavior, and Results [29] (see Table 1). This evaluation framework is ideally suited to a mixed methods approach combining quantitative and qualitative methods, as each level of evaluation requires the implementation of appropriate methodology that is complementary to, but independent of, the other levels. Aspects of the Kirkpatrick Evaluation model have been successfully used in mental health training, for instance in suicide prevention [30].

**Table 1:** Applying the Kirkpatrick Evaluation Model as an evaluation framework for the education and training program.

Level	Key question
Level 1: Reaction	What are the reactions of clinicians to the digital health solution?
Level 2: Learning	Have clinicians learned the relevant knowledge relating to the digital health solution?
Level 3: Behavior	Have clinicians transferred and applied their knowledge of the digital health solution to standard clinical practice?
Level 4: Results	What has been the impact of the digital health solution on service performance, such as efficiency, clinical safety, and clinical outcomes?

The Kirkpatrick Evaluation Model [29] also highlights the importance of ongoing education and training, as the majority of learning is acquired through practice. In order to sustain education and training, the Kirkpatrick model encourages monitoring, reinforcing, encouraging, and rewarding. For this study, monitoring and reinforcing will be completed at 3 months, 6 months, 9 months, and 12 months post-initial education and training, with individual progress reports being provided in order to encourage and reward their adoption of the YMH and Technology Program.

### Measures

#### Participant Profile

Before commencing the education and training program, clinicians will be asked to complete a participant profile. This profile includes questions regarding their educational background, professional background, years of clinical experience, and reasons for participating in the education and training program.

#### Online Seminar, Case Study Webinar, In-Service Workshop Evaluation Form

This form assesses clinician reaction (Level 1) to the education and training program, including satisfaction, engagement, and relevance. This form consists of 20 items, of which the first 15 are rated on a 5‐point Likert scale ranging from “1 = totally disagree” to “5 = totally agree,” and the remaining 5 questions represent an open-ended format. Clinician satisfaction will also be measured using the Training Satisfaction Rating Scale, an existing scale with good content validity [31]. This scale is a 12-item questionnaire assessing participant agreement to objectives and content (eg, “In my opinion, the planned objectives of the [online seminar/ case study webinar/ in-service workshop] were met.”), method and training context (eg, “The [online seminar/ case study webinar/ in-service workshop] enabled us to take an active part in training.”), and usefulness and overall rating (eg, “The education and training received is useful for my personal development.”). In addition to the existing scale, 3 items will be used to evaluate the quality of facilitation (eg, “The facilitator(s) were knowledgeable about the education and training topics*.*”). Lastly, participants will be invited to provide qualitative feedback on the education and training program regarding most liked and disliked aspects, major learnings, and expected results of the training (eg, “What aspects of the training could be improved?”).

#### Knowledge Questionnaire

This questionnaire assesses clinician learning (Level 2) of key concepts of the digital health solution through the online seminars. This questionnaire consists of 30 multiple-choice questions relating to multidimensional assessment and outcomes, clinical staging and illness trajectories, social and biological development, real-time monitoring, and technology-enabled practice. Clinicians must select correct answers out of a list of answers that includes equally plausible distractors. In order to keep the questionnaire brief and reduce the chances of participants learning the answers through repetition, clinicians will be asked to complete 5 randomly selected questions at each time point (ie, at baseline, 3 months, 6 months, 9 months, and 12 months). Furthermore, at each time point, participants will be asked to identify which webinars and case studies they have watched in order to be able to link training engagement with outcomes.

#### Skill Development Scale

This scale assesses confidence in clinical skills (Level 3) central to the BMC Youth Model using 8 visual analogue scales coded as values “0” to “100.” Marks to the left represent low confidence in development of skills, and marks to the right indicate high confidence in development of skills. The 8 items relate to multidimensional assessment, clinical staging, case formulation and treatment planning, shared decision making, intervention skills, outcome monitoring, and care continuity and coordination. Respondents are asked to reflect on each of these areas and rate how well they feel they perform these tasks in clinical practice.

#### Behavior Change Scale

This scale assesses clinician change in behavior (Level 3) using a scale consisting of 9 items that ask how frequently they utilized new knowledge and skills related to multidimensional assessment, clinical staging, intervention matching, shared decision making, monitoring, responding to increased suicidality, and continuity of care. Clinicians rate how often they employed a skill in their standard clinical practice in the past fortnight on a 5-point Likert scale ranging from “1 = not at all (with no clients)” to “5 = always (all of the time, for all clients).” For each item, clinicians can also indicate that the particular skill is not applicable to their service (0 = N/A).

#### Clinical Practice Audit

Change in clinician practice (Level 3) and impact on client outcomes (Level 4) will be assessed using real-time and aggregated data from the InnoWell Platform [32]. Indicators of adoption of the digital health solution include, for example, the frequency with which clinicians access the platform and time spent on the platform, accuracy of clinical staging assignment, and the recommendation of treatment options. Indicators of impact on client outcomes include, for example, efficiency (eg, time to first assessment and wait time for clinical intervention), clinical safety (eg, notifications for suicide thoughts and behaviors and average time to respond to notifications), and clinical outcomes (eg, changes over time in psychological distress as well as social and occupational functioning).

### Procedure

**Figure 1:**
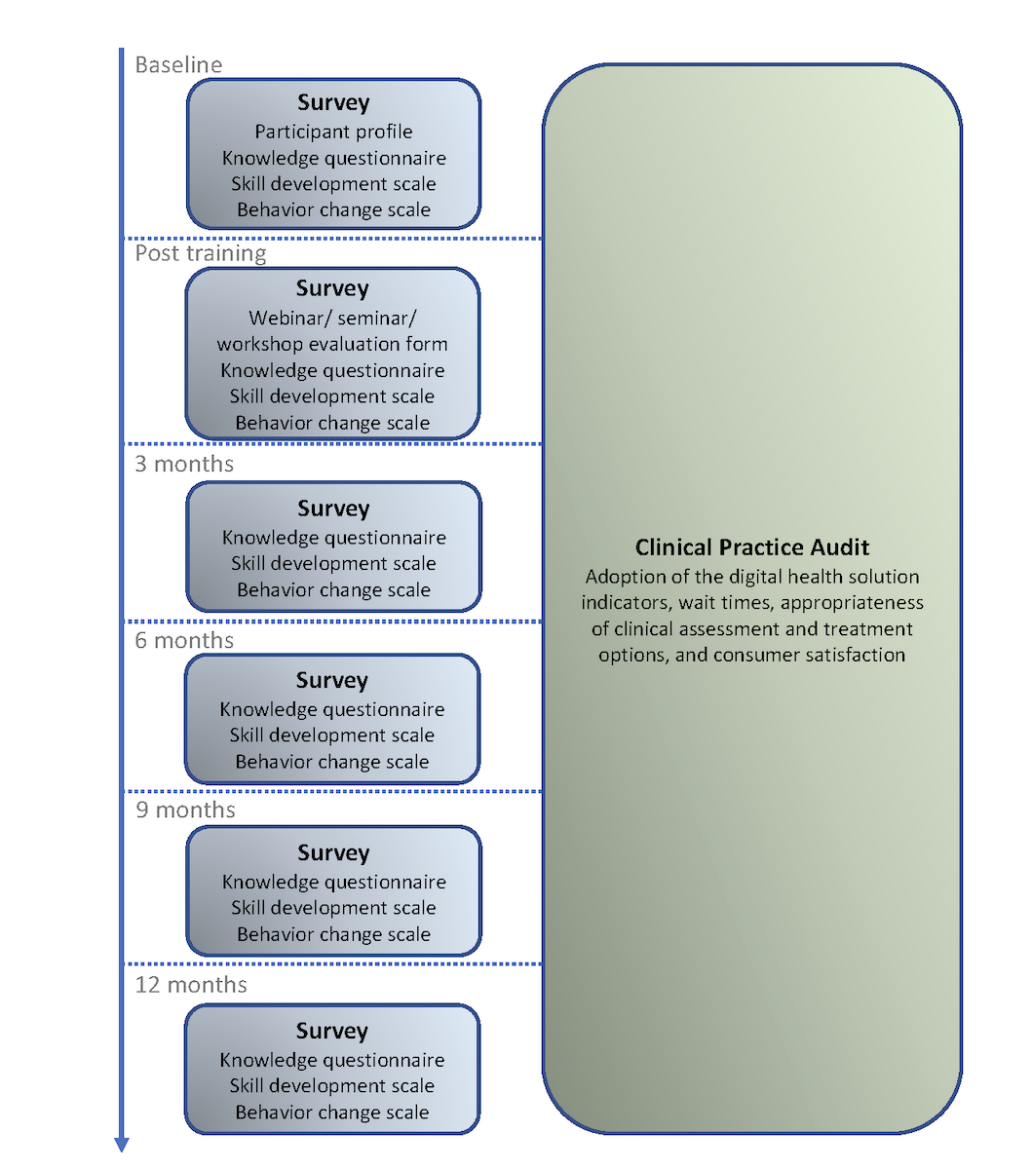
Flow chart of evaluation methods pre- and post-education and training.

The timeline of procedures is summarized in Figure 1. Before commencing the online education and training components, clinicians will complete the participant profile and a baseline knowledge questionnaire, skill development scale, and behavior change scale. A baseline clinical practice audit will also be completed. On completion of the 6 online seminars, the 2 case study webinars, and the in-service workshops, participants will be asked to complete a posttest knowledge questionnaire as well as the skill development and behavior change scales. Importantly, participating clinicians will additionally be contacted after 3 months, 6 months, 9 months, and 12 months and asked to complete the posttest knowledge questionnaire, skill development scale, and behavior change scale as a means to monitor and reinforce knowledge, skills, and behavior [29]. Together with real-time data from the InnoWell Platform (pre-education and training and then every 3 months posteducation and training), participants will privately receive a progress report to encourage and reward their adoption and optimization of the digital health solution [29]. Finally, after each online seminar, case-study webinar, or in-service workshop, clinicians will be asked to complete the evaluation form.

### Participants

Participants will be drawn from 11 participating headspace services in Australia who have previously agreed to implement the HIT. The services include 5 from Sydney, 5 on the North Coast of New South Wales, and 1 in South Australia. Participants will include mental health professionals (eg, psychologists, social workers, mental health nurses, occupational therapists) and service managers. The sample size will be determined by participating services, with all health professionals and service managers being invited to participate in the education and training program.

### Statistical Analyses

In order to assess participants’ reactions to the training, a mixed methods approach will be utilized, specifically a Triangulation Design, Validating Quantitative Data Model [33]. This design would allow the for the quantitative findings (eg, objectives and content, method and training context, usefulness, and quality of facilitation) to be validated and expanded on by including a few open-ended qualitative questions (eg, most liked and disliked aspects, major learnings, and expected results of the training).

Descriptive statistics will be used to analyze quantitative data gathered from the combined evaluation form data, as well as individually for the online seminars, case study webinars, and in-service workshops. A thematic analysis will be conducted to analyze qualitative data collected via the evaluation forms to identify patterns or themes within the data [34]. Procedurally, thematic analysis entails (1) becoming familiar with the data, (2) generating initial codes, (3) searching for themes, (4) reviewing themes, (5) defining and naming themes, and (6) reporting the final thematic concepts. The data will be coded using NVivo by a minimum of 2 different researchers in order to assess interrater reliability. These resulting thematic concepts can then be used to validate and embellish the quantitative survey findings.

In order to assess Learning, Behavior, and Results, pre- and postsurveys and clinical practice audit data will be compared using paired *t* tests to understand short-term impacts, and repeated measures analysis of variance will be used to understand long-term changes in knowledge, skill, behavior, and client outcomes.

### Ethics and Dissemination

Ethics approval has been granted via Sydney Local Health District’s Human Research Ethics Committee (Protocol No X18-0499 & HREC/18/RPAH/715). Research findings will be disseminated through peer-reviewed journals and scientific conference presentations. Participant data will be nonidentifiable and aggregated.

## Results

As of September 2020, clinicians and their services were being recruited to this study. Data collection is expected to be completed and the first results to be submitted for publication in 2021. Results will then be disseminated to the participants, the public, and researchers through publications in journals and presentations at conferences.

## Discussion

This paper details a comprehensive protocol for an evaluation study to assess the effectiveness of the education and training program on the adoption and optimization of use of the digital health solution to provide the knowledge and skills to assess, monitor, and manage the complex needs of young people presenting with mental ill-health problems.

Evaluations of education and training programs within mental health generally focus on the perceived usefulness of such programs, increased knowledge, and confidence in using acquired skills, but frequently neglect assessing the impact on changing clinician knowledge, skills, behaviors, and client outcomes [35]. By using the Kirkpatrick Evaluation Model [29], this study will assess the effectiveness of the education and training program in meeting the learning needs of clinicians (*Reaction*), increasing clinicians’ knowledge of youth mental health (*Learning*), clinicians adopting the digital health solution in standard clinical practice (*Behavior*), and improving service performance (eg, efficiency, clinical safety, and clinical outcomes; *Results*).

While the evaluation protocol will produce valuable insights, there are limitations that should be noted. First, the measures of knowledge and skill rely on self-rating, which are subjective and may be influenced by the participant’s level of self-awareness [36,37]. Second, it is generally recognized as being quite difficult to objectively measure the competence of mental health practitioners [37]. Last, the Kirkpatrick model is not without its flaws; for example, it has received criticism for being overly simplified and lacking an ability to provide information about *how* training can be improved [38]. Nevertheless, the Kirkpatrick model has been successfully utilized in prior mental health training [30] and will provide data in a way that can guide further research.

The research protocol presented here is the first of its kind to explore the education and training of mental health professionals in the use and benefits of technology-enabled solutions. As such, it has the potential to have a significant impact on determining how mental health professionals are trained as technology becomes more integrated into their practice [13,14]. The quantitative and qualitative data obtained from this study will be used to iteratively adapt and improve the education and training program in order to ensure that young people get the right care the first time and to prevent further progression of mental ill-health while in care. These results will also guide the creation of additional education and training resources to keep participating clinicians engaged and to the ensure sustainability of the digital health solution. Future resources may include a case study workbook and the creation of a community of practice that could provide a shared context for practitioners, enabling dialogue between practitioners who might not ordinarily have the chance to interact [39]. Further, our education and training team is currently exploring a more sophisticated online interactive platform to promote ongoing customized learning with participating youth mental health services.

Ultimately, this research will help teach mental health professionals how to facilitate a highly personalized and measurement-based model of care. This may therefore guide clinicians to help young people recover from low functioning associated with subthreshold diagnostic presentations and prevent progression to more serious mental ill-health.

## Abbreviations

BMC: Brain and Mind Centre
HIT: health information technology
YMH: Youth Mental Health
